# Influence of transfusions, hemodialysis and extracorporeal life support on hyperferritinemia in critically ill patients

**DOI:** 10.1371/journal.pone.0254345

**Published:** 2021-07-12

**Authors:** Cornelia Knaak, Friederike S. Schuster, Peter Nyvlt, Patrick Heeren, Claudia Spies, Thomas Schenk, Paul La Rosée, Gritta Janka, Frank M. Brunkhorst, Gunnar Lachmann

**Affiliations:** 1 Department of Anesthesiology and Operative Intensive Care Medicine (CCM, CVK), Charité–Universitätsmedizin Berlin, corporate member of Freie Universität Berlin, Humboldt-Universität zu Berlin, and Berlin Institute of Health, Berlin, Germany; 2 Berlin Institute of Health (BIH), Berlin, Germany; 3 Department of Hematology and Oncology, Universitätsklinikum Jena, Jena, Germany; 4 Klinik für Innere Medizin II, Schwarzwald-Baar-Klinikum, Villingen-Schwenningen, Germany; 5 Clinic of Pediatric Hematology and Oncology, University Medical Center Eppendorf, Hamburg, Germany; 6 Department of Anesthesiology and Intensive Care Medicine, Center for Clinical Studies, Universitätsklinikum Jena, Jena, Germany; Heidelberg University Hospital, GERMANY

## Abstract

**Background:**

Ferritin is the major iron storage protein and an acute phase reactant. Hyperferritinemia is frequently seen in the critically ill where it has been hypothesized that not only underlying conditions but also factors such as transfusions, hemodialysis and extracorporeal life support (ECLS) lead to hyperferritinemia. This study aims to investigate the influence of transfusions, hemodialysis, and ECLS on hyperferritinemia in a multidisciplinary ICU cohort.

**Methods:**

This is a post-hoc analysis of a retrospective observational study including patients aged ≥ 18 years who were admitted to at least one adult ICU between January 2006 and August 2018 with hyperferritinemia ≥ 500 μg/L and of ≥ 14 days between two ICU ferritin measurements. Patients with hemophagocytic lymphohistiocytosis (HLH) were excluded. To identify the influence of transfusions, hemodialysis, and ECLS on ferritin change, multivariable linear regression analysis with ferritin change between two measurements as dependent variable was performed.

**Results:**

A total of 268 patients was analyzed. Median duration between measurements was 36 days (22–57). Over all patients, ferritin significantly increased between the first and last measurement (p = 0.006). Multivariable linear regression analysis showed no effect of transfusions, hemodialysis, or ECLS on ferritin change. Changes in aspartate aminotransferase (ASAT) and sequential organ failure assessment (SOFA) score were identified as influencing factors on ferritin change [unstandardized regression coefficient (B) = 2.6; (95% confidence interval (CI) 1.9, 3.3); p < 0.001 and B = 376.5; (95% CI 113.8, 639.1); p = 0.005, respectively]. Using the same model for subgroups of SOFA score, we found SOFA platelet count to be associated with ferritin change [B = 1729.3; (95% CI 466.8, 2991.9); p = 0.007]. No association of ferritin change and in-hospital mortality was seen in multivariable analysis.

**Conclusions:**

The present study demonstrates that transfusions, hemodialysis, and ECLS had no influence on ferritin increases in critically ill patients. Hyperferritinemia appears to be less the result of iatrogenic influences in the ICU thereby underscoring its unskewed diagnostic value.

**Trial registration:**

The study was registered with www.ClinicalTrials.gov (NCT02854943) on August 1, 2016.

## Introduction

Ferritin is the major iron storage protein located in hepatocytes and the reticulo-endothelial system. As such it regulates iron homeostasis and serves as a marker of total body iron stores [1]. While hypoferritinemia is indicative for depleted body iron storage, hyperferritinemia either occurs with iron overload caused by genetic diseases of iron metabolism, or by excess iron supply via transfusions, or it occurs with disordered iron utilization due to inflammatory conditions [2]. Moreover, elevated ferritin levels are seen in liver and chronic kidney disease, malignancies and metabolic syndrome [3,4]. Hyperferritinemia is a prognostic biomarker as shown for increased 30-day mortality following a hip-fracture [5]. After hematopoietic stem cell transplantation, hyperferritinemia has been linked to decreased long-term survival in pediatric and adolescent patients [6]. Likewise, mortality was increased in critically ill patients with hyperferritinemia [7,8]. Among this patient population, particularly high levels of ferritin have been detected [8]. In critically ill patients, hyperferritinemia is associated with a variety of conditions displaying distinct ferritin levels [8]. In sepsis and septic shock patients, ferritin was most severely elevated with higher levels only detected in patients with hemophagocytic lymphohistiocytosis (HLH) where hyperferritinemia is one of the key laboratory features [8,9]. In HLH patients, ferritin serves as one of the diagnostic criteria [10]. Moreover, it has been shown to be of prognostic value [8]. In the differential diagnosis of anemia, ferritin is one among the essential parameters to evaluate iron deficiency. However, in critical care, procedures such as red blood cell transfusions, hemodialysis and extracorporeal life support (ECLS) are suspected to affect serum ferritin levels thus limiting diagnostic accuracy of ferritin assessment. With each unit of packed red cells, 200–250 mg iron are administered, leading to hyperferritinemia [2,11]. In patients with end-stage renal disease requiring hemodialysis, severely elevated ferritin levels of > 400000 μg/L have been detected [12]. Both hemolysis caused by osmotic and shear stress in the extracorporeal circuit [13] as well as pro-inflammatory immune reactivity triggered by dialysis membranes and catheters [14] might account for this effect. Similar mechanisms apply to suspected hyperferritinemia related to ECLS where hemolysis is a common side effect [15]. Interaction of the immune system with biomaterial prompts an inflammatory reaction including the release of pro-inflammatory cytokines [16]. However, the impact of PRBC transfusion, hemodialysis, and ECLS on ferritin levels and their diagnostic accuracy in critically ill patients remains unclear. Given the multiple confounders of ferritin assessments in the intensive care unit (ICU), our aim was to investigate the influence of transfusions, hemodialysis and ECLS on hyperferritinemia in a multidisciplinary ICU cohort.

## Methods

### Patients and data acquisition

This is a post-hoc analysis of a retrospective observational study [8,17] conducted at the university hospital Charité–Universitätsmedizin Berlin. We included patients aged ≥ 18 years who were admitted to at least one adult surgical, anesthesiological or medical ICU between January 2006 and August 2018 with hyperferritinemia ≥ 500 μg/L and of ≥ 14 days between two ICU ferritin measurements. We chose 14 days at the minimum time interval as it has been demonstrated previously that alterations in ferritin resulting from PRBC transfusion are not seen before two weeks [18]. All ferritin levels were ordered at the discretion of the physicians in charge. As it has been recognized that HLH patients show markedly elevated ferritin levels [8], patients diagnosed with HLH were excluded to rule out a bias towards severe hyperferritinemia. Diagnosis of HLH patients in our cohort was previously described in detail [19]. Patients who were discharged to peripheral wards and readmitted to ICU were excluded. Consequently, only ferritin measurements during ICU stay were considered. In case of more than two ferritin measurements in a single patient, values with maximum time interval between measurements were considered. To estimate iatrogenic iron loss, the number of drawn blood samples including blood gas analyses as well as laboratory tests was counted. Evidence for hemolysis was defined as haptoglobin < 0.3 g/L according to the local reference value [20]. All data including transfusions, hemodialysis, and ECLS were reviewed and retrieved from two electronic patient data management systems operated at the Charité–Universitätsmedizin Berlin (COPRA, Sasbachwalden, Germany and SAP, Walldorf, Germany). Of note, ECLS comprises both extracorporeal lung assist (ECLA) and veno-venous as well as veno-arterial extracorporeal membrane oxygenation (ECMO). To more accurately analyze the influence of ECLS on ferritin levels, we additionally assessed ferritin values while on ECLS between the two measurements. When more than one was available, the highest ferritin level was included for analysis. Diagnosis of sepsis and septic shock was based on their ICD-10 codes: sepsis (A22.7, A39.1, A39.2, A39.3, A39.4, A40.-, A41.-, B37.7, O08.2, O75.3, O85, O88.3, R65.0, R65.1, T80.2, T81.4, T88.0; all without R57.2), septic shock (R57.2), which also has been described previously [8]. If no sequential organ failure assessment (SOFA) score at the day of ferritin measurement was available in the electronic patient charts, it was calculated manually. Change in SOFA score was calculated by the difference between SOFA scores at first and last ferritin measurement. The same applied to change in aspartate aminotransferase (ASAT) to determine extend of liver failure and C—reactive protein (CRP) as a surrogate of inflammation. If no ASAT and CRP measurement was available at the day of ferritin measurement, the closest time point of ASAT and CRP to ferritin assessment was obtained. For clinical reasons, patients were divided into two groups based on ferritin change: ferritin increase versus decrease between two measurements.

### Statistical analysis

Data are expressed as median with (25%-75%) percentiles, or absolute and relative frequencies (n, %), respectively. Differences between groups in terms of continuous parameters were tested using non-parametric Mann-Whitney test for independent groups. Categorical parameters were tested using the Chi-Square-test. Wilcoxon signed-rank test for paired samples was used to compare first and last ferritin measurement, and also first and ECLS measurement. We performed multivariable linear regression analysis with ferritin change between two measurements as dependent variable to investigate influencing factors on ferritin change. Age, sex, body mass index (BMI), diagnosis (others/sepsis/septic shock), number of transfused packed red blood cells (PRBC), change in SOFA score between days of ferritin measurements, number of drawn blood samples, and days during measurements to adjust for different time intervals between measurements were entered into the model as well as days with present hemolysis, change in ASAT and CRP and days of hemodialysis and ECLS. In a post-hoc analysis, scores of each organ system within the SOFA score were entered as independent variables replacing the SOFA score variable. All variables were selected based on clinical and epidemiological relevance. As a sensitivity analysis, we included only patients with at least 28 days between two measurements. Using this cohort, we rerun the first multivariable linear regression analysis while adjusting for the same covariates as before. In another sensitivity analysis, we included only patients with at least 10 transfused PRBC during ICU stay to highlight those with greater transfusion requirements. Finally, we used a multivariable logistic regression model adjusted for age, sex, BMI, days between first and last ferritin measurement, and SOFA score change to evaluate associations between ferritin change and in-hospital mortality. For each model, the unstandardized regression coefficient B and odds ratio (OR), respectively, were reported along with 95% confidence intervals (CI).

### Ethics

Ethics approval was obtained from the institutional review board (Ethikkommission der Charité–Universitätsmedizin Berlin, EA1/176/16). As this is a retrospective study, a waiver of written informed consent was given by the ethics committee. The study was registered with www.ClinicalTrials.gov (NCT02854943) on August 1, 2016.

## Results

### Study population and characteristics

**Fig 1:**
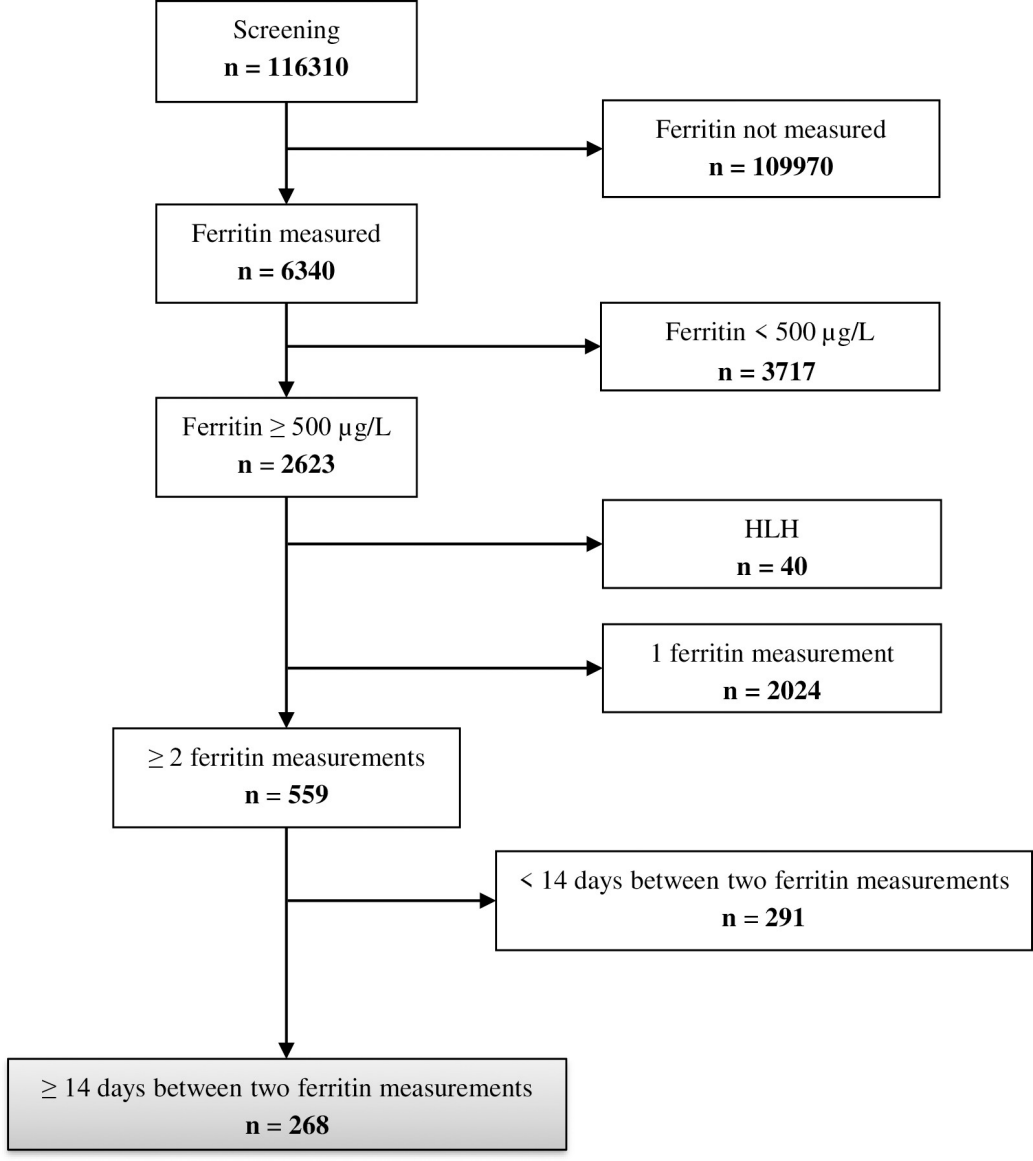
Consort diagram.

116310 patients were admitted to at least one ICU, of which 6340 patients had at least one ferritin value available during their ICU stay. Of these, 2623 had hyperferritinemia (≥ 500 μg/L). After exclusion of 40 patients with HLH [19], 2024 patients with only one ferritin measurement, and 291 patients with < 14 days duration between two ferritin measurements, we finally analyzed 268 patients (Fig 1). Characteristics, ferritin values, and outcome parameters of all analyzed patients are shown in Table 1. A detailed description of all diagnoses over ICU stay of the patients is shown in S1 Table.

**Table 1:** Characteristics, ferritin values and outcome parameters over all patients and between patients with ferritin decrease and increase.

	Analyzed patients (N = 268)	Patients with ferritin decrease(n = 107)	Patients with ferritin increase (n = 161)	P value
Age [years]	59 (44–71)	59 (42–70)	60 (45–71)	0.875^#^
Male sex [n] (%)	162 (60.4%)	66 (61.7%)	96 (59.6%)	0.736^†^
Body Mass Index [kg/m^2^]	25.0 (21.5–29.0)	24.5 (21.2–28.0)	25.0 (21.9–29.0)	0.373^#^
Duration between measurements [d]	36 (22–57)	35 (20–56)	36 (22–60)	0.346^#^
Number of drawn blood samples [n]	270 (155–460)	244 (138–368)	284 (163–481)	0.078^#^
Number of drawn blood samples per day [n]	8 (6–9)	7 (5–9)	8 (6–9)	0.048^#^
Ferritin [μg/L]				
Maximum*	1963 (1264–4842)	1934 (1287–7164)	2085 (1243–4467)	0.734^#^
At first measurement	1298 (784–1410)	1938 (1114–6491)	1007 (574–1797)	< 0.001^#^
At last measurement	1557 (891–2807)	1111 (765–1746)	1896 (1164–3466)	< 0.001^#^
Change between first and last measurement	195 (-373–899)	-584 (-2927 –-239)	706 (260–1601)	< 0.001^#^
Hemolysis**				
Patients with hem olysis [n]	10 (3.7%)	5 (4.7%)	5 (3.1%)	0.507^†^
Days of hemolysis in patients with hemolysis [n]	4 (3–6)	4 (2–5)	5 (4–7)	0.167^#^
ASAT [U/L]				
At first ferritin measurement	40 (23–71)	40 (22–88)	40 (26–62)	0.618^#^
At last ferritin measurement	34 (20–55)	26 (19–47)	38 (22–61)	0.003^#^
CRP [mg/dL]				
At first ferritin measurement	9.5 (4.6–16.8)	11.1 (5.5–17.7)	9.2 (4.3–16.5)	0.198^#^
At last ferritin measurement	5.0 (2.2–9.7)	4.2 (1.8–8.8)	5.4 (2.6–9.9)	0.119^#^
Sepsis without shock [n] (%)*	128 (47.8%)	42 (39.3%)	86 (53.4%)	0.023^†^
Septic shock [n] (%)*	107 (39.9%)	48 (44.9%)	59 (36.6%)	0.179^†^
Transfusion**				
PRBC, patients [n] (%)	233 (86.9%)	92 (86.0%)	141 (87.6%)	0.704^†^
PRBC per transfused patient [n]	10 (5–20)	8 (4–16)	12 (6–24)	0.012^#^
Hemodialysis**				
Patients with hem odialysis [n]	72 (26.9%)	28 (26.2%)	44 (27.3%)	0.834^†^
Days of hemodialy sis per dialyzed patients [n]	14 (6–31)	10 (5–20)	18 (6–33)	0.109^#^
ECLS**				
Patients with ECLS [n]	10	3 (2.8%)	7 (4.3%)	0.514^†^
Days of ECLS in patients with ECLS [n]	17 (5–29)	5 (2 –n.a.)	21 (8–27)	0.360^#^
SOFA score				
ICU admission*	7 (3–11)	7 (4–11)	6 (3–11)	0.947^#^
Maximum*	14 (11–17)	14 (10–17)	14 (11–17)	0.927^#^
Maximum**	12 (9–16)	11 (8–15)	12 (9–16)	0.296^#^
At first ferritin measurement	8 (5–11)	8 (5–11)	7 (4–11)	0.125^#^
At last ferritin measurement	4 (2–8)	4 (2–6)	4 (2–8)	0.138^#^
ICU duration [d]*	67 (33–101)	65 (30–98)	69 (34–102)	0.639^#^
In-patient duration [d]*	96 (67–143)	98 (65–153)	95 (68–139)	0.569^#^
Deceased [n]*	78 (29.1%)	26 (24.3%)	52 (32.3%)	0.158^†^

*Continuous quantities in median with quartiles; P values were calculated using Mann-Whitney-U test*^*#*^ *and χ2 test*^*†*^, *respectively*. *ASAT*, *aspartate aminotransferase; CRP*, *c-reactive protein; ECLS*, *extracorporeal life support; ICU*, *Intensive Care Unit; n*.*a*., *not applicable; PRBC*, *packed red blood cells; SOFA*, *Sequential organ failure assessment*.

*Analyzed over ICU stay.

**Only time between ferritin measurements considered.

### Influence of transfusions, ECLS, and hemodialysis on ferritin levels

**Fig 2:**
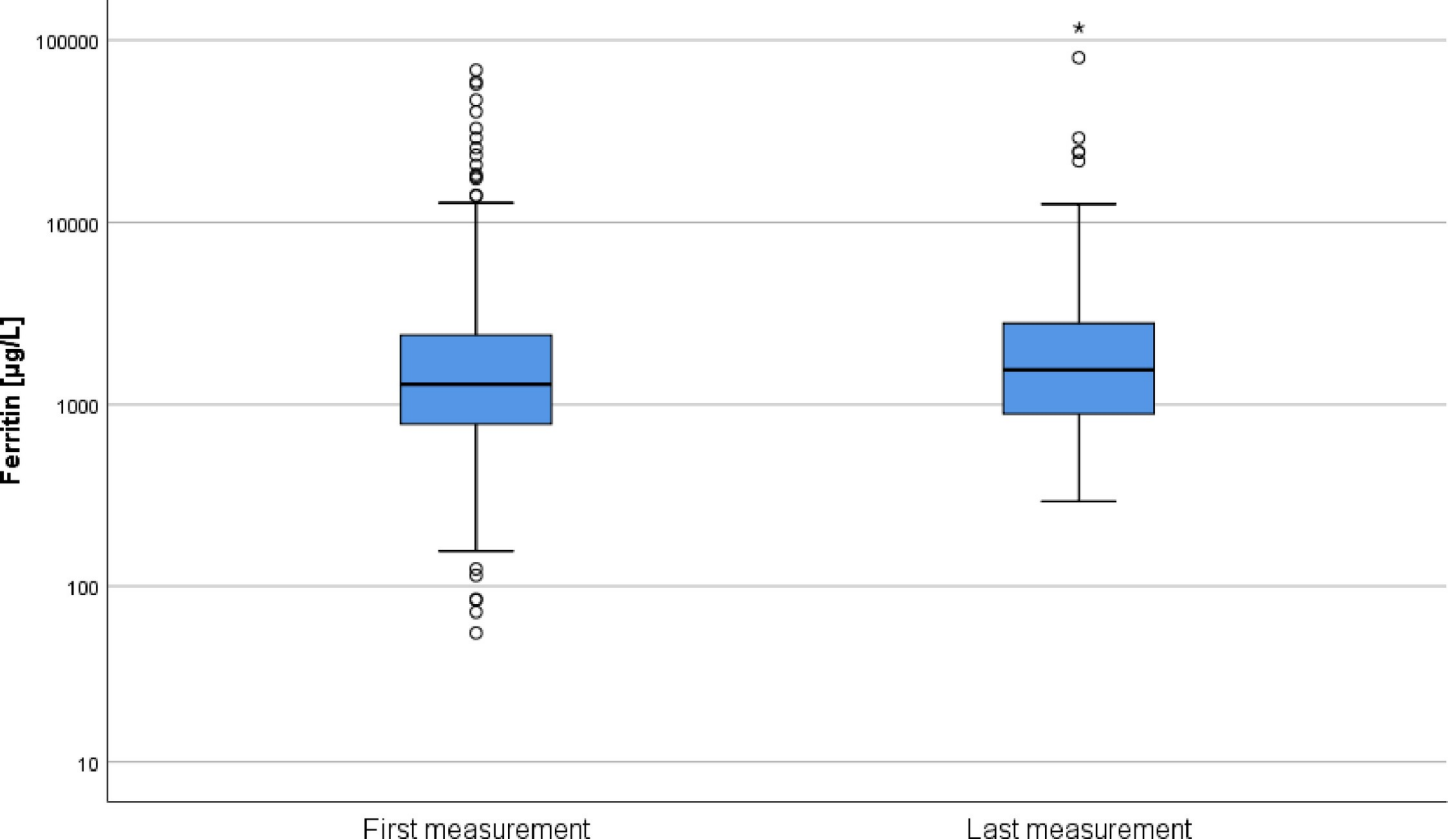
Distribution of ferritin values between first and last measurement. Median duration between two measurements was 36 (22–57) days. Boxplots show median with quartiles (25%, 75%). Whiskers below and above show minimum and maximum values, respectively. For visualization, y-axis is plotted in logarithmic scale.

Over all patients, ferritin significantly increased between the first and last measurement (p = 0.006; Table 1 and Fig 2). When transfusions, hemodialysis, and ECLS were considered separately in univariate analyses, we found a significant increase of ferritin in PRBC transfused patients (Table 2). However, multivariable linear regression analysis showed no effect of transfusions, hemodialysis, and ECLS on ferritin change (Table 3). Of note, we found a significant influence of ASAT change and SOFA score change on ferritin increase. Using the same model for subgroups of SOFA score, we found SOFA platelet count to be associated with ferritin change [B = 1729.3 (95% CI 466.8, 2991.9); p = 0.007; S2 Table]. Sensitivity analysis of patients with at least 28 days between two measurements (n = 177) showed no significant influence of transfusions, hemodialysis or ECLS on ferritin change. Same results occurred in another sensitivity analysis of patients with at least 10 transfused PRBC (n = 126). Seven out of ten ECLS patients had a ferritin value available while on ECLS (between the first and last ferritin measurement). However, we did not find any significant ferritin increase between first and ECLS ferritin measurement in these patients (p = 0.310).

**Table 2:** First and last ferritin measurement between different groups.

	Number of patients	First ferritin measurement	Last ferritin measurement	P value between first and last measurement
Patients with PRBC transfusion	233 (86.9%)	1331 (817–2486)	1594 (934–2831)	0.015
Patients without PRBC transfusion	35 (13.1%)	1102 (669–1876)	1021 (659–2628)	0.207
Patients with hemodialysis	196 (73.1%)	1509 (826–3697)	1695 (1099–3896)	0.117
Patients without hemodialysis	72 (26.9%)	1232 (772–2175)	1473 (857–2603)	0.026
Patients with ECLS	10 (3.7%)	1229 (763–2522)	2143 (1041–3141)	0.139
Patients without ECLS	258 (96.3%)	1298 (780–2413)	1552 (885–2825)	0.012

*Continuous quantities in median with quartiles; P values were calculated using Wilcoxon signed-rank test for paired samples*. *ECLS*, *extracorporeal life support; PRBC*, *packed red blood cells*. *For all groups*, *only time between ferritin measurements was considered*. *P values between first and last measurement in each row were calculated using Wilcoxon signed-rank test for paired samples*.

**Table 3:** Multivariable linear regression analysis for ferritin change between first and last ferritin measurement.

Covariates	Regression coefficient B	95% CI	P value
Age	-74.6	-152.8, 3.5	0.061
Sex (male)	-1070.1	-3774.7, 1634.4	0.437
BMI	93.1	-67.6, 253.9	0.255
SOFA score change	376.5	113.8, 639.1	0.005
Days during measurements	-3.1	-78.5, 72.3	0.936
Number of drawn blood samples	3.2	-8.1, 14.5	0.583
Days of hemolysis	-835.7	-2794.4, 1123.1	0.402
ASAT change	2.6	1.9, 3.3	< 0.001
CRP change	25.0	-32.9, 83.0	0.395
Diagnoses*	-559.4	-2570.7, 1452.0	0.584
Number of transfused PRBC	-18.1	-100.0, 63.9	0.664
Days of hemodialysis	3.7	-109.7, 117.1	0.949
Days of ECLS	-96.0	-432.2, 240.3	0.575

*Multivariable linear regression analysis was performed with ferritin change between first and last ferritin measurement as dependent variable (R^2^ = 0*.*225; All variance inflation factors < 7)*. *ASAT*, *aspartate aminotransferase; BMI*, *Body mass index; CI*, *confidence interval; CRP*, *c-reactive protein; ECLS*, *extracorporeal life support; PRBC*, *packed red blood cells; SOFA*, *Sequential organ failure assessment*.

*Others/sepsis/septic shock.

### Ferritin change and in-hospital mortality

In univariate analyses over all patients, ferritin change significantly differed between survivors and non-survivors (median 120 μg/L versus median 493 μg/L; p = 0.007). However, this was not confirmed in a multivariable logistic regression model adjusted for age, sex, BMI, days between first and last ferritin measurement and SOFA score change (p = 0.797).

## Discussion

This is the first study to demonstrate that transfusions, hemodialysis, and ECLS had no impact on ferritin levels in critically ill patients treated in ICU. Change in ASAT and SOFA score were identified as influencing factors on ferritin increase. In clinical practice, ferritin allows for the evaluation of body iron stores as it is sensitive to even slight iron deficiency [2]. In addition to inflammation, infection, liver and chronic kidney diseases, malignancies and metabolic syndrome as well as hereditary iron overload, and repetitive PRBC transfusions lead to increased ferritin levels [21]. In fact, Gao et al. [22] found ferritin levels to be increased in patients after repeated PRBC transfusions, i.e. > 50 units over one year. Also, Froissart et al. [23] detected an increase in ferritin after PRBC transfusion, yet without affecting the diagnostic accuracy. In our study, patients with PRBC transfusion compared to those without had an increased median ferritin during their ICU stay while non-transfused patients experienced a decrease in ferritin. Of note, a total of 86.9% of all patients received PRBC compared to 13.1% who received no PRBC. However, we saw no impact of PRBC transfusions on ferritin in multivariable analysis. Even in patients who had received ten or more units of PRBC, no relevant change in ferritin levels was seen. In their prospective study of 61 critically ill patients, Boshuizen et al. [24] observed unchanged ferritin levels after transfusion of one unit of PRBC. It appears that other factors that influence ferritin values in the critically ill mask the effect of PRBC transfusions, even if given as frequently as in our study. One reason that PRBC transfusions had no influence on ferritin levels might be the rather high number of drawn blood samples in the ICU. As a result of frequent blood drawing, iron is continuously removed from the body leading to iatrogenic anemia prompting PRBC transfusions. By repetitive PRBC transfusions, this loss may be balanced leaving ferritin levels unchanged. In our analyses, the dominant factors for ferritin increase were the changes in SOFA score and in ASAT levels. These findings implicate that the degree of organ failure, particularly liver function determines ferritin levels. With each unit increase in ASAT, ferritin rose by 2.6 μg/L. As liver impairment is assessed within the SOFA score, we sought to differentiate scores of each organ system. Here, we found low platelet count to be linked to ferritin change. Given that thrombocytopenia is frequently seen in liver failure, due to splenic sequestration as a result of portal hypertension and decreased thrombopoietin synthesis [25], liver cell necrosis appears to be one of the driving factors of ferritin change. However, thrombocytopenia also occurs in multiple bone marrow disorders and might therefore not exclusively be linked to liver failure. Conversely, bone marrow suppression can also be caused by liver diseases including viral hepatitis and alcoholic liver disease [25]. Moreover, thrombocytopenia is indicative for coagulopathies such as disseminated intravascular coagulation. We therefore suggest that also coagulation disorders may be an important factor of the association between SOFA score and hyperferritinemia.

Hemolytic anemias are generally associated with a rise in ferritin [26]. Increased ferritin levels have also been observed after hemolysis in patients receiving hemodialysis [27]. However, hemolysis in the context of hemodialysis resulting from shear stress in the extracorporeal circuit, chloramine contamination, hyperthermic or hypoosmolar dialysate has become a rather rare event [28]. Moreover, rises in ferritin would have only occurred if hemolysis was severe enough to induce anemia prompting increased iron absorption or even PRBC transfusions, thereby leading to increased ferritin. In our analysis, no influence of hemodialysis on ferritin levels could be detected. We therefore assume that hemodialysis in today’s critical care setting does not affect the diagnostic value of ferritin. Our findings were similar with regard to ECLS: Ferritin levels remained unchanged in the presence of ECLS. It has been demonstrated previously that along with the use of extracorporeal devices comes an increased rate of hemolysis [15] and an inflammatory response [16]. However, hemolysis was detected in only 3.7% of our cohort. Given this rare event, the effect on ferritin in this cohort might be negligible. The inflammatory reaction in response to extracorporeal treatment also appears to have less of an influence on ferritin.

In the critical care setting, uncertainty remains of the diagnostic value of ferritin, as hyperferritinemia is frequently seen in multiple conditions in the ICU [8]. There was concern that transfusions might impair diagnostic reliability of ferritin measurement in anemia [23]. Where hyperferritinemia was attributed to transfusions and organ replacement therapies, the significance of hyperferritinemia might have been underestimated. With respect to the results of the present study, we believe that elevated ferritin levels should not be misinterpreted as iatrogenic hyperferritinemia, at least in critically ill patients, where liver failure seems to be the major drive of hyperferritinemia. Nevertheless, patients with multiple transfusions who are at risk for treatable iron overload, should be followed by ferritin measurements after discharge from the ICU.

It is important to note that hyperferritinemic syndromes with extreme disease inherent ferritin levels such as in HLH or macrophage activation syndrome-HLH (MAS-HLH) are not part of this analysis. Hence, conclusions from this analysis drawing our attention to liver failure as major trigger for hyperferritinemia must not neglect a minority of patients, where extreme ferritin levels may indicate HLH or macrophage activation syndrome-HLH (MAS-HLH) [8].

Ferritin has been found to be an independent predictor of in-hospital mortality [8]. In the present analysis, we saw higher ferritin changes in non-survivors than in survivors. However, ferritin change did no longer remain statistically significant in multivariable analysis suggesting that hyperferritinemia itself is related to risk of death while an increasing ferritin is not.

Our study bears several important limitations. First, as this is a retrospective analysis, we depended on available data of ferritin measurements performed during ICU stay. Thus, it cannot be ruled out that patients with relevant ferritin courses have remained undetected. Second, time intervals differed between measurements. Third, our study period of twelve years is rather long. It cannot be ruled out that practice changes affecting our results have occurred during this time. Fourth, sepsis and septic shock were defined retrospectively using ICD-10 codes, although a prospective assessment following the Surviving Sepsis Campaign criteria might have revealed more accurate results. Finally, we did not adjust for underlying conditions which might have determined ferritin levels. However, this was on purpose as we aimed to detect influencing factors on ferritin change where underlying conditions were present at both time points.

## Conclusions

In this study, we could demonstrate that transfusions, hemodialysis, and ECLS had no influence on ferritin increases in critically ill patients. Hyperferritinemia appears to be less of a cause of iatrogenic influences in the ICU thereby underscoring its unskewed diagnostic value. Changes in ASAT and SOFA score, however, were the major drivers of ferritin increase in critically ill patients without HLH. Hence, hyperferritinemia in critically ill patients should be interpreted in the context of possible liver impairment.

## Supporting information

S1 Table Diagnoses during ICU stay. (DOCX)

S2 Table Multivariable linear regression analysis for ferritin change between first and last ferritin measurement. (DOCX)

## Abbreviations

ASAT: Aspartate aminotransferase
BMI: Body Mass Index
CI: Confidence interval
CRP: C-reactive protein
CT: Computed tomography
ECLS: Extracorporeal life assist
Hb: Hemoglobin
HLH: Hemophagocytic lymphohistiocytosis
ICD: International classification of diseases
ICU: Intensive care unit
MAS: Macrophage Activation Syndrome
mmol/L: Mmoles/liter
NK cell: Natural killer cell
OR: Odds ratio
Plt: Platelets
PRBC: Packed red blood cells
SOFA: Sequential organ failure assessment
U: Units
