# Supplementary material for: Influence of transfusions, hemodialysis and extracorporeal life support on hyperferritinemia in critically ill patients
Source: PLoS One. 2021 Jul 12;16(7):e0254345. doi: 10.1371/journal.pone.0254345 (PMC8274924; doi:10.1371/journal.pone.0254345)
Supplement: S1 Table — (DOCX) [file pone.0254345.s001.docx]

**Table S1. Diagnoses during ICU stay.**

|  | **Analyzed patients  (N = 268)** | **Patients with ferritin decrease (n = 107)** | **Patients with ferritin increase (n = 161)** | **P value** |
| --- | --- | --- | --- | --- |
| Tuberculosis [n] (%) | 4 (1.5 %) | 3 (2.8 %) | 1 (0.6 %) | 0.149 |
| Hepatitis [n] (%) | 22 (8.2 %) | 14 (13.1 %) | 8 (5.0 %) | 0.018 |
| VZV [n] (%) | 7 (2.6 %) | 2 (1.9 %) | 5 (3.1 %) | 0.534 |
| HSV [n] (%) | 17 (6.3 %) | 4 (3.7 %) | 13 (8.1 %) | 0.154 |
| Influenza [n] (%) | 11 (4.1 %) | 4 (3.7 %) | 7 (4.3 %) | 0.805 |
| Acute or chronic liver disease [n] (%) | 92 (34.3 %) | 40 (37.4 %) | 52 (32.3 %) | 0.391 |
| History of stem cell/organ transplantation [n] (%) | 36 (13.4 %) | 17 (15.9 %) | 19 (11.8 %) | 0.337 |
| CMV [n] (%) | 17 (6.3 %) | 7 (6.5 %) | 10 (6.2 %) | 0.913 |
| HIV [n] (%) | 7 (2.6 %) | 4 (3.7 %) | 3 (1.9 %) | 0.346 |
| EBV [n] (%) | 10 (3.7 %) | 4 (3.7 %) | 6 (3.7 %) | 0.996 |
| Acute or chronic renal disease [n] (%) | 211 (78.7 %) | 84 (78.5 %) | 127 (78.9 %) | 0.941 |
| Bacterial, viral or fungal infection [n] (%) | 265 (98.9 %) | 105 (98.1 %) | 160 (99.4 %) | 0.342 |
| Inflammation without infection [n] (%) | 109 (40.7 %) | 42 (39.3 %) | 67 (41.6 %) | 0.700 |
| Autoimmune disease [n] (%) | 25 (9.3 %) | 10 (9.3 %) | 15 (9.3 %) | 0.994 |
| Solid malignancy [n] (%) | 33 (12.3 %) | 14 (13.1 %) | 19 (11.8 %) | 0.754 |
| Hematologic malignancy [n] (%) | 24 (9.0 %) | 12 (11.2 %) | 12 (7.5 %) | 0.291 |
| Pre-existing immunosuppression [n] (%) | 77 (28.7 %) | 34 (31.8 %) | 43 (26.7 %) | 0.369 |

*Diagnoses are based on the International Classification of Diseases, 10^th^ Revision (ICD-10) codes as previously described in (8). Due to various numbers of ICD codes in each single patient, patients might overlap between the disease groups. P values calculated using the χ2 test. CMV: Cytomegalovirus; EBV: Epstein-Barr virus; HIV: Human immunodeficiency virus; HSV: Herpes simplex virus; VZV: Varicella-zoster virus.*
