# Supplementary material for: Influence of transfusions, hemodialysis and extracorporeal life support on hyperferritinemia in critically ill patients
Source: PLoS One. 2021 Jul 12;16(7):e0254345. doi: 10.1371/journal.pone.0254345 (PMC8274924; doi:10.1371/journal.pone.0254345)
Supplement: S2 Table — (DOCX) [file pone.0254345.s002.docx]

**Table S2. Multivariable linear regression analysis for ferritin change between first and last ferritin measurement.**

| Covariates | Regression coefficient B | 95 % CI | P value |
| --- | --- | --- | --- |
| Age | -71.5 | -149.7, 6.7 | 0.073 |
| Sex (male) | -1166.4 | -3859.7, 1527.0 | 0.395 |
| BMI | 102.4 | -58.3, 263.1 | 0.211 |
| SOFA paO_2_/FiO_2_ | -162.3 | -1061.3, 736.8 | 0.723 |
| SOFA Platelets | 1729.3 | 466.8, 2991.9 | 0.007 |
| SOFA Bilirubin | 773.2 | -484.4, 2030.7 | 0.227 |
| SOFA Hypotension | 725.1 | -155.0, 1605.3 | 0.106 |
| SOFA GCS | -119.6 | -925.1, 685.9 | 0.770 |
| SOFA Creatinine | 112.5 | -734.7, 959.8 | 0.794 |
| Days during measurements | 12.9 | -64.7, 90.5 | 0.744 |
| Number of drawn blood samples | 0.3 | -11.3, 12.0 | 0.955 |
| Days of hemolysis | -731.3 | -2682.3, 1219.6 | 0.461 |
| ASAT change | 2.5 | 1.8, 3.3 | < 0.001 |
| CRP change | 18.5 | -39.6, 76.5 | 0.532 |
| Diagnoses* | -320.8 | -2339.7, 1698.1 | 0.755 |
| Number of transfused PRBC | -16.3 | -98.3, 65.8 | 0.697 |
| Days of hemodialysis | 36.8 | -79.5, 153.1 | 0.534 |
| Days of ECLS | -74.1 | -411.2, 263.0 | 0.665 |

*Multivariable linear regression analysis was performed with ferritin change of two measurements as dependent variable (R² = 0.252; All variance inflation factors < 7). ASAT, aspartate aminotransferase; BMI, Body mass index; CRP, C-reactive protein; ECLS, extracorporeal life support; F_i_O_2_, Fraction of inspired oxygen; GCS, Glasgow come scale; p_a_O_2_, partial pressure of oxygen in arterial blood; PRBC, packed red blood cells; SOFA, Sequential organ failure assessment. *Others/sepsis/septic shock.*
